# Implant-Based Strategies for Interdental Papilla Preservation and Regeneration in Single-Tooth Edentulous Spaces of the Esthetic Zone: A Narrative Review

**DOI:** 10.3390/dj14100667

**Published:** 2026-10-13

**Authors:** Andrei Macris, Sergiu Drafta, Alexandru Eugen Petre

**Affiliations:** Prosthetic Department, Faculty of Stomatology, “Carol Davila” University of Medicine and Pharmacy, 020021 Bucharest, Romania; andrei.macris@umfcd.ro (A.M.); alexandru.petre@umfcd.ro (A.E.P.)

**Keywords:** interdental papilla, papilla regeneration, esthetic dentistry, implant esthetics, connective tissue graft, pink esthetic score

## Abstract

**Background/Objectives**: Loss of the interdental papilla in a single-tooth edentulous site within the esthetic zone is a frequent clinical issue with functional and psychosocial impact. This literature review aimed to appraise and synthesize the published clinical evidence on implant-based strategies for regenerating and preserving the interdental papilla in single-tooth edentulous spaces within the esthetic zone, with emphasis on methodological quality, esthetic outcomes over time, and patient satisfaction. **Methods**: A structured search was performed across PubMed/MEDLINE and Scopus, complemented by gray-literature sources including Semantic Scholar, complemented by gray-literature sources, resulting in 498 papers. The studies included adult patients with single-tooth edentulism in the anterior maxilla, reported at least one esthetic parameter, and followed up patients for a minimum of 6 months. Data analysis included study characteristics, surgical and prosthetic techniques, esthetic results, complications, and patient satisfaction. **Results**: A total of 27 studies fulfilled the inclusion criteria. The short-term studies (<12 months) showed favorable interdental papilla fill with immediate flapless implant placement followed by provisional restoration. Mid-term results (12–36 months) indicated stable interdental papilla presence, alongside Pink Esthetic Score/White Esthetic Score values with ridge preservation and augmentation increasing predictability. In the long-term studies (>36 months), minimal recession was reported after 12 months. **Conclusions**: Immediate implant placement with provisional restoration, minimally invasive approaches, and connective tissue grafting in thin gingival phenotypes are the strategies most consistently associated with favorable interdental papilla preservation outcomes in the available observational evidence; causal efficacy under controlled conditions remains to be established by adequately powered randomized trials.

## 1. Introduction

The loss of the interdental papilla (IP) in edentulous spaces within the esthetic zone is a considerable concern for clinical treatment, with both functional and psychosocial implications. The lack of the IP results in the presence of black triangles, which have negative consequences on smile esthetics and quality of life [1,2,3]. Furthermore, the absence of the IP can make phonetics difficult and lead to food impaction, causing discomfort and difficulties in oral hygiene [4].

Esthetic reconstruction of the IP has become more and more important in contemporary dentistry, owing to patients who are demanding a natural and stable prosthetic or implant solution [5,6]. In the last 20 years, different treatment options have been developed which combine surgical and prosthetic procedures aimed at maintaining or regenerating the IP. Of these, immediate implant placement [7,8] with a provisional restoration has shown significant predictability in preserving the gingival form, especially in areas possessing a thick gingival phenotype and a buccal bone plate [1,6,7]. In addition, the distance of ≤5 mm from the contact point to the bone crest (or missing IP) has been invariably associated with almost total presence of IP [2,4].

The socioeconomic consequences of these results could be considered impressive. In such cases, patients with unfavorable results often complain of reduced self-confidence and satisfaction, and their social life and professional performance could be influenced [3]. Conversely, successful treatments that can stabilize the IP and produce high esthetic scores of PES (Pink Esthetic Score) or WES (White Esthetic Score) for more significant social integration versus patients’ perceived redistribution issues regarding oral health [9].

However, the available evidence currently reported is limited due to small sample sizes, short follow-up periods and heterogeneity of study designs that makes it impossible for a standardization of clinical guidelines [8,10]. Furthermore, the attainment of an ideal esthetic result is difficult and adverse esthetic results may even arise with favorable cases [3].

Given this scenario, the aim of the present literature review is to appraise and synthesize the published clinical evidence of techniques used for regenerating and preserving the IP in single-tooth edentulous spaces within the esthetic zone, with emphasis on esthetic outcomes over time, patient satisfaction, and the methodological quality of the available evidence. Non-implant papilla management strategies—including prosthetic simulation with ovate pontic design, soft-tissue grafting around natural abutment teeth, and stand-alone orthodontic extrusion without subsequent implant placement—fall outside the scope of this review.

## 2. Materials and Methods

### 2.1. Literature Identification and Selection

**Figure 1:**
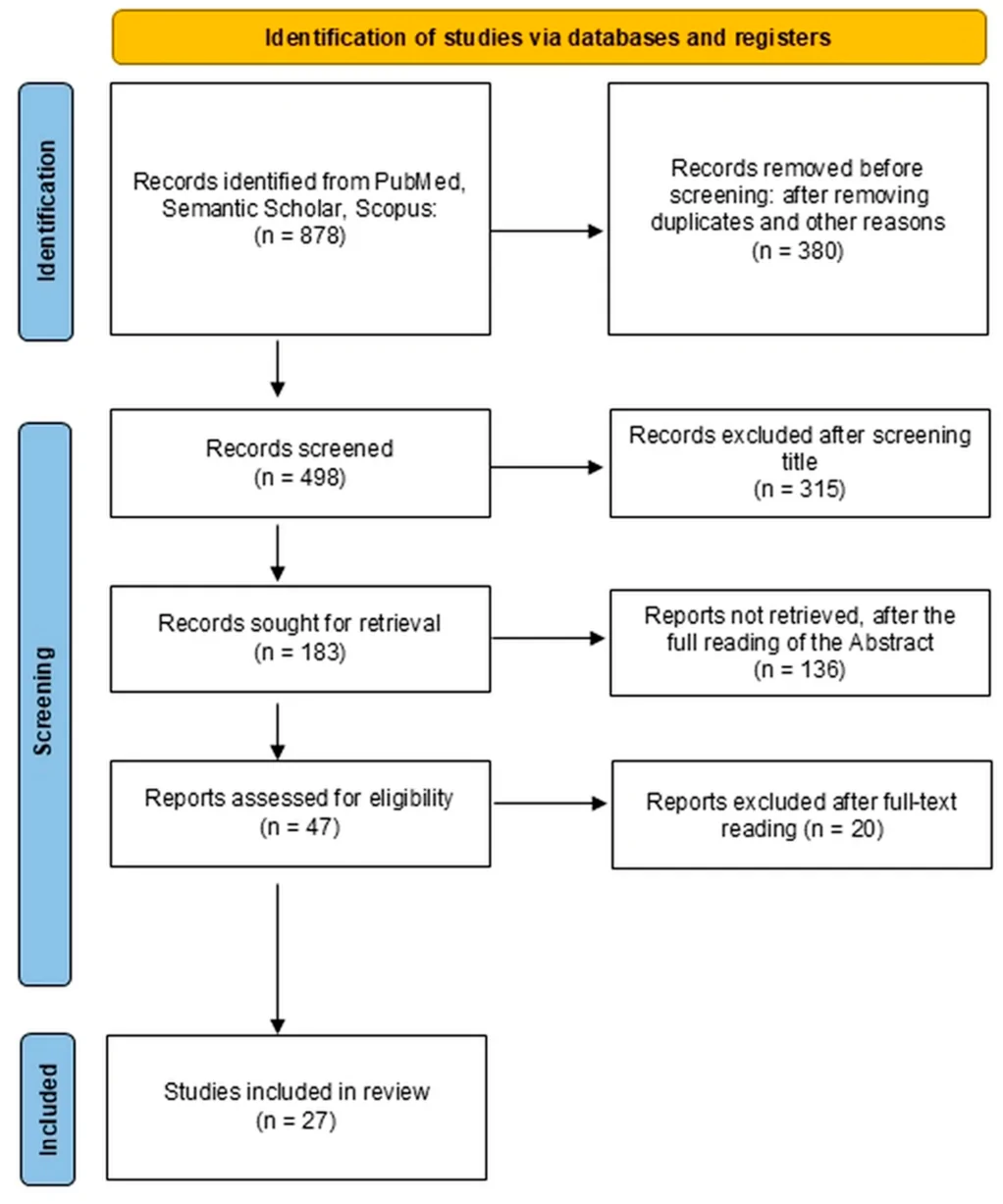
The diagram illustrates the identification, screening, eligibility assessment, and inclusion process for studies considered in the literature review.

This literature review was structured as a narrative synthesis of the available clinical evidence on techniques for the esthetic restoration, preservation, or regeneration of the IP in single-tooth edentulous spaces. The review did not aim to quantify treatment effects or rank interventions statistically, but to organize the evidence by clinical approach, follow-up interval, esthetic outcome, and patient-reported relevance. The identification and progressive selection of records are summarized in Figure 1, which provides a transparent overview of the literature-screening process used to define the evidence base included in the review. The review is classified as a narrative synthesis rather than a systematic or scoping review for the following reasons: (1) the synthesis is organized thematically around clinical approach and follow-up interval rather than a single focused PICO question; (2) the search, while structured and reproducible, was not preregistered; (3) no formal meta-analytical pooling or intervention hierarchy was produced; and (4) the critical appraisal of included studies is a descriptive, non-validated quality rating rather than a domain-based risk-of-bias assessment. Where PRISMA-related language or flow diagram elements have been adopted, they serve transparency purposes only and do not imply full systematic review methodology.

Title/abstract screening was performed independently by two authors (AM, SD) using the pre-defined eligibility criteria (Table 1). Full-text articles identified as potentially eligible after title/abstract screening were then retrieved and assessed independently by the same two authors. Discrepancies at both stages were resolved by discussion; when consensus could not be reached, the third author (AEP) acted as the deciding arbiter. The reasons for exclusion of full-text articles were recorded and are reported in the literature selection flow diagram (Figure 1).

### 2.2. Search Strategy

A structured search was performed across two primary electronic databases—PubMed/MEDLINE and Scopus—conducted up to and including December 2025. These databases were selected for their complementary coverage of the indexed dental and biomedical literature: PubMed/MEDLINE provides controlled-vocabulary MeSH-term indexing of core biomedical journals, while Scopus offers broad title-abstract-keyword indexing across more than 22,000 peer-reviewed journals, encompassing the dental implant literature covered by EMBASE for this topic area. The database search was complemented by manual screening of reference lists of included studies and a supplementary keyword search in Semantic Scholar, used as a gray-literature aggregator to identify conference abstracts, preprints, and cross-disciplinary sources not indexed in the primary databases. The search focused on studies reporting clinical methods for restoring or preserving the IP in single-tooth edentulous spaces within the esthetic zone. The search was limited to English language and human studies. The Boolean search strategy combined terms related to IP, esthetic dentistry, connective tissue grafting (CTG), Pink Esthetic Score, IP regeneration, and implant esthetics. The following search strings were applied:

- PubMed/MEDLINE:

((“interdental papilla”[tiab] OR “papilla regeneration”[tiab] OR “papilla reconstruction”[tiab] OR “black triangle”[tiab] OR “papilla fill”[tiab]) AND (“dental implant”[MeSH Terms] OR “dental implants”[tiab] OR “single implant”[tiab] OR “single-tooth implant”[tiab] OR “implant-supported”[tiab] OR “connective tissue graft”[tiab] OR “soft tissue augmentation”[tiab]) AND (“esthetic zone”[tiab] OR “anterior maxilla”[tiab] OR “esthetic outcome”[tiab] OR “pink esthetic score”[tiab] OR “PES”[tiab] OR “papilla index”[tiab])) AND (“humans”[MeSH Terms]) AND (“English”[Language])

- Scopus:

TITLE-ABS-KEY ((“interdental papilla” OR “papilla regeneration” OR “papilla reconstruction” OR “black triangle” OR “papilla fill”) AND (“dental implant” OR “single implant” OR “single-tooth implant” OR “connective tissue graft” OR “soft tissue augmentation”) AND (“esthetic zone” OR “anterior maxilla” OR “pink esthetic score” OR “papilla index” OR “PES” OR “WES”)) AND (LIMIT-TO (LANGUAGE, “English”))

- Semantic Scholar:

Searches performed using the following term combinations: “interdental papilla” + “dental implant”; “papilla regeneration” + “esthetic zone”; “single tooth implant” + “papilla” + “esthetic outcome”; “connective tissue graft” + “papilla” + “single implant”.

After removal of duplicates and records excluded for other reasons, 498 citations were screened for relevance, and the steps are summarized in the literature selection flow diagram (Figure 1).

The search strategy was developed through iterative discussion among the authors to ensure a clinically relevant balance between sensitivity and specificity. The mentioned keyword combinations and Boolean operators were refined through pilot searches and then adapted to the indexing structure of each database. Article selection emphasized clinical relevance to anterior single-tooth edentulous spaces, the presence of IP-related or esthetic outcomes, and sufficient follow-up to permit interpretation of soft-tissue stability.

### 2.3. Scope of the Included Literature

Articles were retained when they involved adult patients with single-tooth edentulism in the maxillary esthetic zone and reported surgical, prosthetic, orthodontic, or combined interventions intended to maintain, restore, or regenerate the IP. The principal outcomes of interest included IP height, IP fill, IP index values, PES/WES, gingival margin stability, presence or absence of black triangles, patient satisfaction, and esthetic complications.

Studies addressing only animal or in vitro models, exclusively posterior sites, multiple adjacent missing teeth without a clearly identifiable single-tooth component, or very small clinical series with limited interpretability were not retained for synthesis. The minimum follow-up considered clinically useful was 6 months, because IP morphology and peri-implant soft-tissue remodeling require an initial healing interval before meaningful esthetic interpretation can be made.

In summary, studies were assessed for eligibility against pre-defined inclusion and exclusion criteria, presented in Table 1 below. The criteria were established prior to data extraction and guided by a PICO-structured framework adapted for narrative reviews.

The selection process was therefore used to construct a focused, clinically interpretable body of evidence rather than a formal hierarchy of intervention effectiveness. Studies addressing immediate implant protocols and soft-tissue esthetic outcomes were included when they contributed data on IP preservation or peri-implant esthetics [1,5,6,7,11]. Retrospective evidence was also considered when IP levels adjacent to single implants were specifically assessed [2]. Comparative or subgroup data were retained when they clarified the influence of CTG, graft material, or orthodontic extrusion on IP behavior [10,12,13].

### 2.4. Data Organization

Data from the selected articles were extracted independently by two authors (AM, SD) using a pre-specified, standardized extraction form developed a priori. The form captured: study design and Oxford Centre for Evidence-Based Medicine (OCEBM) level; sample size and recruitment setting; implant timing and placement protocol; flap or flapless approach; provisionalization protocol; CTG and augmentation procedures; orthodontic interventions; PES/WES values; IP index scores; gingival margin changes; reported complications; and patient-reported satisfaction. Discrepancies between the two extractors were identified through cross-checking and resolved by discussion, with the third author (AEP) providing the deciding judgment when consensus was not reached. Missing or unclear data were recorded as not reported rather than imputed, and none of the authors of the 27 studies were contacted.

When individual articles reported more than one intervention arm or subgroup, the relevant subgroup information was maintained to support comparison within the narrative synthesis. For example, Yoshino et al. [10] compared immediate implant placement with and without CTG, Lédo et al. [12] reported findings related to autogenous and synthetic grafting, and Kan et al. [13] evaluated orthodontic extrusion as an IP-regenerative approach.

### 2.5. Critical Appraisal Framework

Each included study was appraised independently by two authors (AM, SD) across the following dimensions, with disagreements resolved by consensus with the third author (AEP):

- Study design and evidence level, classified according to the Oxford Centre for Evidence-Based Medicine (OCEBM) 2011 levels: Level 1b (randomized controlled trial), Level 2b (prospective comparative cohort), Level 3b (retrospective or cross-sectional comparative study), and Level 4 (prospective or retrospective non-comparative case series or observational study).
- Sample size adequacy, with studies enrolling fewer than 15 patients flagged as carrying a substantive risk of type II error.
- Enrollment strategy, distinguishing consecutive or criteria-based recruitment (lower selection bias) from convenience or non-specified recruitment (higher selection bias).
- Outcome measurement, distinguishing validated and standardized instruments (PES/WES, Nordland–Tarnow classification [14], quantitative linear measurements) from non-standardized or clinician-defined assessments.
- Follow-up completeness, considering both the declared follow-up duration and the adequacy of dropout reporting.
- Presence of a comparator group (parallel-arm or intra-patient control).

On the basis of these six dimensions, each study was assigned a Methodological Quality Rating (MQR), defined as a descriptive summary judgment rather than a validated domain-based risk-of-bias score:

- MQR-A (High methodological quality): prospective RCT with adequate sample size, pre-specified outcomes, and complete reporting.
- MQR-B (Acceptable methodological quality): RCT with minor design limitations, or prospective controlled study with pre-defined protocol.
- MQR-C (Moderate methodological quality): prospective non-comparative cohort or non-randomized comparative study with notable design limitations.
- MQR-D (Limited methodological quality): retrospective study, single-arm case series, or observational cohort with substantive design limitations.

This rating is not equivalent to a formal risk-of-bias assessment and should not be interpreted as such. No validated risk-of-bias instrument (e.g., Cochrane RoB 2, Newcastle-Ottawa Scale) was applied, as the heterogeneity of study designs across this narrative review precludes the uniform application of a single validated tool. The MQR is provided to assist readers in contextualizing individual study findings and is presented in Table 2.

Because a narrative synthesis rather than a meta-analysis is presented, the appraisal was used to contextualize individual study findings within the Results section and to qualify the strength of evidence underlying clinical recommendations in the Discussion section, rather than to exclude studies on quality grounds alone.

Across the 27 included studies, two studies were classified at OCEBM Level 1b (randomized controlled trials), five at Level 2b (prospective comparative cohorts), four at Level 3b (retrospective or cross-sectional comparative studies), and sixteen at Level 4 (non-comparative prospective or retrospective case series or observational studies). Overall methodological quality was rated MQR-B for two studies (Yosino et al. [10] and Cardaropoli et al. [15]), MQR-C for twelve studies, and MQR-D for thirteen studies. No study met criteria for MQR-A. The predominance of small non-comparative single-arm cohorts (*n* < 30 in 16 of 27 studies) and the near-absence of randomized controlled evidence restrict the certainty of conclusions that can be drawn. These methodological limitations are considered throughout the narrative synthesis and are extensively addressed in the limitations of the evidence discussion below.

## 3. Results

The 27 studies fulfilling the inclusion criteria are summarized in Table 2 (study characteristics and critical appraisal).

Important interpretive note: All numerical values cited in the Results sections that follow (IP fill rates, recession measurements, PES/WES scores, patient satisfaction figures) are study-specific observations derived from individual study populations with heterogeneous designs, surgical protocols, implant systems, outcome definitions, and follow-up intervals. These values cannot be directly compared across studies and do not constitute pooled estimates. Ranges cited across multiple studies (e.g., recession from −0.04 mm to −0.29 mm) reflect the span of individual-study means, not a meta-analytically derived distribution. Readers should interpret these figures as illustrative of the clinical patterns observed within each specific study context rather than as benchmarks applicable to general clinical practice.

### 3.1. Short-Term Outcomes (<12 Months)

The Pink Esthetic Score (PES; maximum 14 points comprising seven parameters—mesial IP, distal IP, curvature of the facial mucosa, level of the facial mucosa, root convexity, and soft-tissue color and texture, each scored 0–2) was the primary composite esthetic index across most short-term studies. A PES ≥ 12 is considered a high esthetic outcome; values of 8–11 are generally acceptable; scores < 8 represent clinically perceptible esthetic deficiencies. The White Esthetic Score (WES; maximum 10 points: tooth form, volume/outline, color, surface texture, and translucency, each 0–2) complements the PES with ≥8 as the threshold for satisfactory crown esthetics. IP fill was classified throughout the review according to the Nordland–Tarnow system: Class 0 = complete fill to the contact point; Class I = partial fill, not reaching the contact point; Class II–III = absent IP below or at the cementoenamel junction.

Within prospective cohorts and comparative studies, short-term results were consistent in demonstrating that minimally invasive immediate protocols—principally flapless insertion with immediate provisional restoration and selective soft-tissue augmentation—achieve satisfactory IP and soft-tissue contours at 12 months. Early studies established that a contact point–crestal bone distance of ≤5 mm is strongly associated with complete or near-complete IP fill (Class 0 or high Class I), a threshold now consistently reproduced across subsequent evidence [2]. When this distance exceeds 5 mm, IP fill falls from near-complete to approximately 50%, underscoring the anatomical determinism of this measurement.

Immediate flapless protocols reported IP fill values ≥ 50% (Class 0 or I, i.e., visually present IP) in 70–90% of sites at 6–12 months [6,16]. It should be noted that this figure encompasses both complete fill (Class 0) and partial fill (Class I); complete fill was not universally achieved, and residual incomplete IP accounted for a proportion of these ‘positive’ outcomes. However, Class I IP—in which the IP is visually present but does not reach the contact point—is generally considered esthetically acceptable at conversational distance. Midfacial tissue levels were highly stable, with mean recession ranging from −0.04 mm to −0.29 mm—values that fall below the 0.5 mm threshold of clinical perceptibility and confirm preservation of midfacial architecture in the short term [1,6,17].

Gingival phenotype significantly modulated short-term outcomes. Thin phenotypes exhibited greater soft-tissue instability—including higher rates of midfacial recession and more variable IP fill—compared with thick phenotypes, which consistently demonstrated superior IP maintenance and more predictable midfacial contour preservation [18]. This phenotype-dependent vulnerability is biologically plausible given the reduced tissue volume and keratinized width of thin phenotypes, which are proposed to make them more susceptible to surgical trauma and post-operative recession; however, this mechanistic explanation is inferred from observational associations rather than established by controlled mechanistic studies. Thin gingival phenotypes therefore represent a high-risk category requiring supplementary soft-tissue augmentation.

The sole RCT directly addressing soft-tissue augmentation in this context—Yoshino et al. [10]—documented a midfacial change of −0.25 ± 0.11 mm in the CTG group versus −0.70 ± 0.18 mm without grafting at 12 months. This 0.45 mm difference was statistically significant (*p* < 0.001) and carries direct clinical meaning: the no-graft recession value of −0.70 mm exceeds the 0.5 mm clinical significance threshold, placing it in the range of visually perceptible and clinically meaningful soft-tissue loss, whereas the grafted sites remained below this threshold. This finding provides the strongest individual-study support for the potential benefit of CTG in patients with a thin gingival phenotype undergoing immediate implant protocols; it represents a single small RCT (*n* = 10 per arm) and should be interpreted as preliminary evidence requiring confirmation rather than establishing a routine clinical indication.

In sites treated with a modified pedicle CTG around delayed implants, both mesial and distal IP heights increased by approximately 1.5–2.0 mm by 6 months [19]. Given that the normal IP measures approximately 4–5 mm in total height, a 1.5–2.0 mm gain represents 30–40% recovery of the missing IP volume, a clinically meaningful improvement particularly at compromised sites where conventional surgical access is limited.

PES values in short-term case series typically averaged 11–12 at 12 months, corresponding to the ‘high esthetic’ category (PES ≥ 12) or the upper range of ‘acceptable’ (PES 8–11) by established criteria [3,7]. However, one cohort (Raes et al. [7]) reported a notable 24% esthetic failure rate (PES < 8 and/or WES < 6) at 12 months—meaning nearly one in four patients fell below the minimally acceptable esthetic threshold despite technically successful implant integration—a finding that warrants careful interpretation (see Section 3.4). Despite this, patients consistently reported high satisfaction, suggesting a partial dissociation between objective scoring and subjective experience at this time-point.

Key short-term signs: Regular IP fill (≥50%) with immediate/flapless implant placement and provisionalization [6,16], gingival phenotype-dependent reaction [17], contact point–bone crest distance as a significant determinant [2], and early benefits of targeted soft-tissue grafting in carefully selected delayed sites [19].

### 3.2. Mid-Term Outcomes (1–3 Years)

Between 12 and 36 months, the included studies consistently reported stabilization of IP levels and midfacial soft-tissue contours, with implant survival rates approaching or reaching 100% and PES/WES values in the acceptable-to-high range across most cohorts. However, important nuances emerge when individual study findings are interpreted in the context of established scoring thresholds.

Yoshino et al. [10], the only RCT addressing soft-tissue augmentation in single-tooth immediate protocols, confirmed at 12 months that CTG placement at the time of immediate implant insertion conferred a statistically significant midfacial advantage (−0.25 mm vs. −0.70 mm; *p* < 0.001), with the no-graft arm exhibiting clinically significant recession that persisted into the mid-term follow-up. This finding reinforces the short-term data and suggests that the benefit of soft-tissue grafting, once established, is maintained over the 12-month period rather than diminishing with time.

Two prospective series by Meijndert et al. [20,21] reported median PES values of six (2020) and seven (2021), alongside WES values of eight in both. It must be noted explicitly that median PES values of six and seven fall below the generally accepted ≥8 threshold for minimally acceptable peri-implant esthetic outcome, and does not qualify as ‘acceptable’ by the criteria of Belser et al. [3], which are widely used in the field. This sub-threshold finding is in contrast with the more favorable PES values (11–12) reported by cohorts using immediate provisional protocols; the discrepancy likely reflects differences in implant design (bone-level tapered configuration), site preparation approach (ridge preservation in defect sockets), and the earlier emergence of the provisional restoration rather than an immediate soft-tissue conditioning profile. Patient satisfaction in the 2021 cohort reached 86.6% on VAS (0–100 scale)—a high subjective rating that, when read alongside the below-threshold PES, illustrates the partial dissociation between objective esthetic scoring and patient perception in the mid-term window.

Cardaropoli et al. [15]—the highest-quality RCT in this review (*n* = 48; OCEBM Level 1b)—directly compared immediate placement to delayed placement preceded by ridge preservation at 12 months. No statistically significant difference in PES, WES, or midfacial recession was found between groups, with both achieving scores in the acceptable-to-high esthetic range. This controlled finding is clinically meaningful: it indicates that, when supported by effective ridge preservation, delayed implant placement can produce esthetic outcomes equivalent to immediate placement. For clinicians and patients, this expands the range of predictable approaches available in the esthetic zone beyond the immediate protocol alone.

Palattella et al. [22] found no statistically significant difference in IP levels or facial contours at 24 months between immediate replacement and immediate restoration, suggesting that both prosthetic protocols preserve IP architecture equivalently when site selection criteria are met. In the autogenous vs. synthetic graft comparison (Lédo et al. [12]), a high proportion of sites achieved a favorable IP score at 12 months regardless of graft material, indicating that synthetic substitutes may be biomechanically sufficient for IP support at the mid-term horizon, with potential benefits in terms of reduced donor-site morbidity. Furthermore, two additional series confirmed that immediate esthetic zone implant placement and restoration preserved peri-implant soft-tissue dimensions and crown form across the mid-term horizon, reporting favorable esthetic outcomes at 18 months [23] and at 12–27 months follow-up [24], respectively.

In aggregate, mid-term evidence supports the stability of IP outcomes established in the short term, particularly with immediate protocols and CTG in thin gingival phenotypes. However, the below-threshold PES values observed in the Meijndert et al. [20,21] studies serve as a reminder that not all immediate or delayed protocols produce uniformly high esthetic scores, and that implant design, site morphology, and augmentation strategy are independent determinants of esthetic performance at this time-point.

Primary mid-term indicators: Sustained IP presence and satisfactory PES/WES values at 1–24 months following immediate protocols, enhanced in thin gingival phenotypes by CTG [10,12,15,16,25].

### 3.3. Long-Term Outcomes (>3 Years)

Long-term follow-up data (>36 months), available in only five studies [8,9,13,21,26]—of which four were single-arm observational or retrospective designs—suggest that the principal soft-tissue changes occur within the first 6–12 months of function, after which IP levels and midfacial mucosa appear to undergo minimal further alteration. This observation, while consistent across the available evidence, should be interpreted cautiously given the small number of long-term studies, the absence of controlled comparisons, and the heterogeneity of implant protocols and follow-up intervals represented.

Arora et al. [8] reported that in a cohort of immediately placed and restored implants followed for 24–60 months, mean midfacial mucosal recession stabilized at approximately 0.3 mm over the full follow-up interval. This value falls below the 0.5 mm clinical perceptibility threshold, confirming that the long-term trajectory of midfacial soft-tissue change is sub-clinical in magnitude and does not compromise the visible esthetic outcome for the majority of sites. The 24–60-month range in Arora et al. also implies heterogeneous follow-up durations within the cohort; however, the mean recession was consistent across the range, supporting a stable tissue plateau after the initial remodeling period.

Amato et al. [26] demonstrated that dehiscence sites treated with immediate buccal-tilted subcrestal implant placement combined with both hard- and soft-tissue augmentation maintained esthetic levels over 24–60 months, with a mean PES of 12.5—a value in the high esthetic range (≥12) that indicates near-ideal peri-implant soft-tissue quality was preserved even at sites where baseline anatomy was compromised by a facial dehiscence defect. This finding suggests that rigorous augmentation at the time of implant placement can offset the inherent esthetic risk associated with dehiscence sites over the long term.

The most comprehensive long-term dataset was provided by Baer et al. [9]—the only multicenter prospective 5-year cohort in this review (*n* = 77)—who employed a 4-grade IP index (Grade 0 = absent; Grade 1 = present but incomplete; Grade 2 = complete fill to the contact point; Grade 3 = overgrowth). At 60 months, 90.1% of sites achieved Grade 2 or 3 (‘optimal’ IP fill), representing complete or near-complete interproximal tissue fill. This high proportion, maintained from earlier follow-up time-points without evidence of progressive deterioration, constitutes the most robust long-term evidence for IP stability following immediate provisionalization on a tapered conical implant. PES also improved progressively from baseline to 60 months, confirming a trajectory of long-term esthetic maturation rather than decline.

Orthodontic extrusion (Kan et al. [13]) produced a mean IP gain of approximately 4 mm, maintained over a mean follow-up of 48.4 months (range 24–72 months). To contextualize this gain: the normal IP measures approximately 4–5 mm in total height, meaning that ~4 mm of gain corresponds to near-complete reconstruction of the missing IP—an outcome that conventional surgical or prosthetic approaches cannot reliably deliver when vertical tissue deficit exceeds 2–3 mm. The proposed biological mechanism—coronal repositioning of the periodontal ligament and supracrestal tissue complex through controlled tooth extrusion, followed by an osseous resection step—is understood to drive new interproximal tissue formation; this mechanism is biologically consistent with periodontal extrusion physiology but has not been confirmed by histological or controlled studies in this specific clinical context. The long-term stability of this gain (maintained without significant deterioration at 24–72 months) supports orthodontic extrusion as a predictable option in selected cases with severe papillary insufficiency.

Key long-term signals: Long-term evidence supports the durability of esthetic outcomes achieved through immediate implant protocols, with soft-tissue recession stabilizing below clinical significance thresholds after the first year, high rates of complete IP fill maintained at 5 years, and orthodontic extrusion offering uniquely large IP gains in surgically challenging cases [8,9,13,26].

A summary of published esthetic outcomes, types of complications and patients satisfaction at different times of follow-up is reported in Table 3 and illustrates the concordance and heterogeneity of results between clinical protocols.

### 3.4. Reported Complications and Esthetic Shortfalls

Across the 27 included studies, biological failure rates (implant loss, peri-implantitis requiring removal) were consistently low or absent. However, esthetic shortfalls—defined as failure to reach established PES or IP score thresholds—were more prevalent and clinically informative. Implant survival should not be equated with esthetic success; the findings below make this distinction explicit.

Raes et al. [7] reported the highest esthetic failure rate in the cohort: 24% of cases failed to reach PES ≥ 8 and/or WES ≥ 6 at 12 months—the minimum thresholds defining clinically acceptable esthetic outcomes on scales of 0–14 and 0–10, respectively. This means that approximately one in four patients, despite technically successful implant integration, presented with esthetic deficiencies perceptible to both the clinician and, in most cases, the patient. The associated 8% clinical complication rate in this cohort comprised esthetic complications (notably soft-tissue recession, crown color discrepancy, or visible transition at the implant shoulder) rather than biological failures. The higher-than-average failure rate in Raes et al. [7] is partly attributable to the inclusion of patients with thin gingival phenotypes and anatomically challenging sites without systematic supplementary CTG use; when these anatomical risk factors are present, esthetic failure rates rise substantially.

Papillary recession in the remaining cohorts was generally of low clinical magnitude. Most series reported recession in the range of tenths of a millimeter (0.1–0.5 mm), which falls at or below the 0.5 mm perceptibility threshold. At one year, early soft-tissue changes were predominantly associated with thin phenotype and inadequate buccal bone support rather than with specific surgical technique [5,6,11]. In mid-term cohorts, average recession in the order of 0.06–0.29 mm was reported [8,15,27], consistent with stable biological remodeling rather than progressive loss. These values confirm that when patient selection and augmentation are appropriate, papillary recession is a manageable rather than inevitable consequence of esthetic zone implant treatment.

The collective evidence identifies three key modifiable risk factors for esthetic shortfall: thin gingival phenotype (which increases both recession risk and IP fill variability), deficient or dehisced buccal bone plate (which predisposes to midfacial tissue loss without augmentation), and a contact point–crest distance exceeding 5 mm (which predictably reduces IP fill to ≤50%). Addressing these factors through gingival phenotype-appropriate grafting, ridge preservation, and prosthetic design optimization represents the primary preventive strategy for esthetic complications.

### 3.5. Patient-Reported Satisfaction

Patient-reported outcomes, where measured, paralleled but did not mirror objective esthetic scores. Satisfaction was assessed using the Visual Analog Scale (VAS; 0–100, where ≥80 is broadly consistent with high satisfaction) in most reporting studies, and values above this threshold were consistently observed across short- and mid-term cohorts.

Meijndert et al. [21] reported a mean VAS satisfaction of 86.6% at 12 months in the ridge preservation and delayed implant cohort, a result in the ‘high satisfaction’ range despite a median PES of 7—which, as discussed in Section 3.2, falls below the ≥8 minimally acceptable esthetic threshold. De Rouck et al. [17] reported 93% patient satisfaction at 12 months in a consecutive immediate implant cohort, despite a mean midfacial recession of 0.29 mm—a value near the upper boundary of clinical perceptibility. In the longest-follow-up study (Baer et al. [9]), patient-reported satisfaction improved progressively over 5 years alongside objective PES improvement, suggesting a parallel maturation of both subjective and objective esthetic outcomes in the long term.

These high satisfaction rates must be interpreted in light of the documented discordance between patient-reported and clinician-assessed esthetic outcomes. Raes et al. [7] reported a 24% objective esthetic failure rate (PES < 8 and/or WES < 6) alongside high patient satisfaction—a paradox that is not unique to implant dentistry and reflects three well-recognized phenomena: (1) patients evaluate their outcome relative to their pre-treatment status rather than against an absolute esthetic ideal, making even incomplete IP restoration subjectively rewarding; (2) patients show lower sensitivity to sub-millimeter soft-tissue and IP level differences than trained clinical assessors; and (3) VAS instruments in their standard form capture global satisfaction rather than component-specific esthetic concerns, introducing a ceiling effect that obscures item-level dissatisfaction.

The clinical implication is that patient satisfaction, though a valid outcome, should not be used as a surrogate for esthetic success in reporting or decision-making. Validated clinician-assessed indices (PES/WES; IP index; quantitative linear measurements) remain necessary to detect esthetic shortfalls that patient self-report may not capture, particularly when outcomes are communicated in the context of clinical guidelines or referral pathways.

## 4. Discussion

### 4.1. Principal Findings

This review shows that reconstruction and preservation of the IP in the single-tooth edentulous space remains a significant and unresolved esthetic and functional clinical challenge. Of the studies evaluated, immediate implant placement with provisional restorations was the most extensively studied approach and the one most consistently associated with favorable IP height and gingival contour outcomes across the available evidence [5,6,7]. Favorable outcomes were persistently reported with minimally invasive techniques, especially flapless insertion, and in the presence of intact buccal bone and a thick gingivalphenotype.

Although in most cases the IP demonstrated improvement or maintenance of presence over 1 year, variation in surgical approach, patient gingival phenotype, and prosthodontic treatment plan led to a wide range of esthetic outcomes. Long-term studies also indicate that most soft-tissue changes are seen in the first 6–12 months, as after this period, IP and midfacial mucosa tend to stabilize for 24–60 months [8,9].

### 4.2. Immediate vs. Delayed Implant Placement

Immediate versus delayed placement: Several non-randomized studies reported higher IP fill and esthetic scores in association with immediate loading—particularly when augmented with CTG or ridge preservation—compared with delayed placement; however, the absence of randomization in the majority of these comparisons means that residual confounding by site selection, anatomy, and operator preference cannot be excluded, and the observed differences should be interpreted as associative rather than causal [3,11,15].

### 4.3. Role of CTG and Gingival Phenotype

Soft-tissue augmentation: There was an increase in IP height and PES values with CTG in both immediate and delayed implant-placement protocols [10,19]. Extended pedicle CTG was associated with a partial IP gain at 6 months in a single small case series [18], an observation consistent with the proposed role of soft-tissue volume in supporting interproximal tissue height at compromised sites. At 12 months, potentially similar or improved soft-tissue stability was indicated with synthetic grafts compared with autogenous grafts [12].

Thick gingival phenotypes were associated with enhanced IP fill and reduced recession, whereas thin phenotypes were associated with greater soft-tissue instability and higher rates of midfacial recession in the observational data. These associations are biologically plausible given the reduced keratinized tissue volume of thin phenotypes, but cannot be regarded as established causal relationships in the absence of prospective controlled studies with phenotype as the primary stratification variable [6,10,18].

### 4.4. Contact Point–Crest Distance and Buccal Bone Integrity

Contact point–crestal bone distance: A mean critical threshold of ≤5 mm was highly associated with the presence of an IP, consistent with studies that first identified this concept [2,4].

Buccal bone preservation: A preserved buccal bone plate is a prerequisite for predictable esthetic outcomes at immediately placed implant sites. At sites presenting with a facial dehiscence defect, recession is substantially more likely in the absence of hard- and soft-tissue augmentation [8,26]. Flapless versus flapped approach: Flapless approaches showed minimal marginal recession and better IP stability when contrasted against flapped process [1,7].

Graft material: There is some evidence that synthetic substitutes might have graft stability in soft tissue similar to that of autogenous grafts, with reduced morbidity [12].

**Figure 2:**
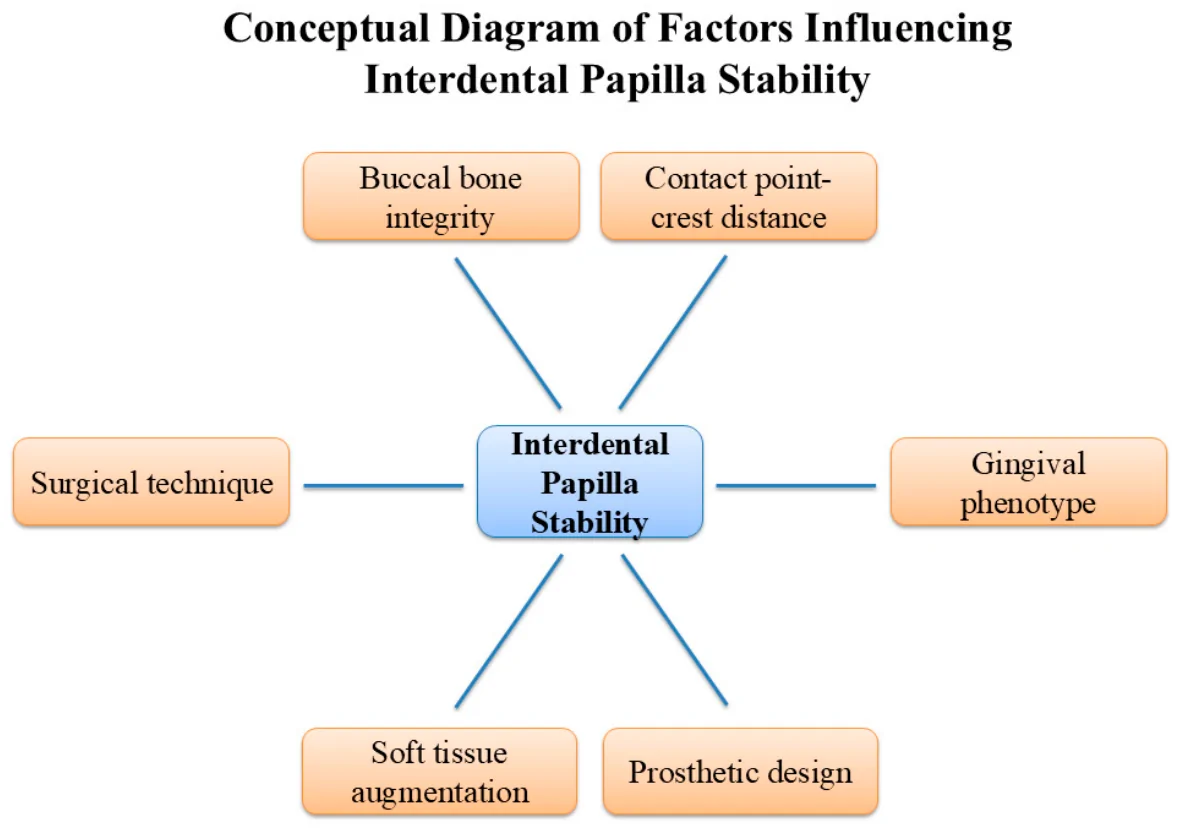
Conceptual diagram illustrating the main biological and technical factors that influence IP stability around single-tooth restorations in the esthetic zone.

To better conceptualize the main variables, the main biological and technical factors involved in IP stability are schematically presented in a diagrammatic manner by drawing mutually interacting circles, consisting of tissue phenotype, surgical approach and prosthetic design (Figure 2).

IP morphology is determined by a multifactorial interaction between buccal bone integrity, contact point–crest distance, gingival phenotype, the surgical technique employed, the extent of soft-tissue augmentation, and prosthetic design. These domains collectively contribute to the predictability of IP preservation or regeneration following implant-based or restorative treatment.

### 4.5. Long-Term Stability and Special Approaches

Orthodontic extrusion: Where there is severe loss of the IP, orthodontic extrusion was an effective substitute that produced an average IP gain of 4 mm was obtained, with stability confirmed during long-term follow-up [13]. This illustrates its place in certain high-risk individuals where traditional implant-based procedures are inadequate.

Bone augmentation and ridge preservation: Ridge preservation and contour augmentation has been shown to improve soft-tissue contours around implants or reduce the risk of recession. Bone ridge augmentation also showed promise but has not been widely supported [28].

### 4.6. Patient-Reported Outcomes

Some studies incorporated subjective esthetic evaluations. Overall patient satisfaction was very high, and the changes in PES/WES followed self-reported improvements [3,21]. Certain cohorts reported significantly high esthetic failure rates (24%) even after technically successful treatment, demonstrating that clinician-measured indices and patient expectations do not match [7].

### 4.7. Limitations and Research Priorities

The critical appraisal of the 27 included studies reveals several important methodological limitations that constrain the certainty of the evidence synthesized in this review.

- First, the level of evidence is predominantly low. Only two RCTs were identified (Yoshino et al. [10]; Cardaropoli et al. [15]), representing 7.4% of the evidence base. Both were single-center studies with small samples per arm (*n* = 10 and *n* = 24, respectively), limiting their statistical power to detect treatment differences. The remaining 25 studies were observational or non-comparative in design, with 16 classified at OCEBM Level 4. Conclusions derived from this evidence base must therefore be interpreted as reflecting consistent clinical patterns rather than established treatment hierarchies.
- Second, sample sizes are frequently insufficient. Sixteen of the 27 studies enrolled fewer than 30 patients, and eight enrolled fewer than 15. At these sample sizes, individually observed esthetic outcomes—including PES/WES values, IP fill rates, and recession measurements—carry wide confidence intervals that are rarely reported, limiting the generalizability of individual findings.
- Third, selection MQR is a pervasive risk. In the majority of non-randomized studies, the method of patient recruitment is not specified. Most series describe selected, protocol-compliant patients with favorable baseline anatomy (thick gingival phenotype, intact buccal plate), potentially overestimating the predictability of immediate implant protocols in routine clinical practice.
- Fourth, outcome measurement is heterogeneous. Esthetic outcomes were measured using validated instruments (PES/WES, Nordland–Tarnow index, standardized digital photography) in approximately half of the included studies, while the remainder employed non-standardized clinical assessments, visual analog scales, or qualitative descriptors. This limits direct comparison across studies and makes pooled interpretation unreliable.
- Fifth, follow-up duration is generally short. Seventeen of 27 studies had a maximum follow-up of 12 months or less. Only five studies provided follow-up exceeding 36 months (Cosyn et al. [5]: 36 months; Arora et al. [8]: 24–60 months; Amato et al. [26]: 24–60 months; Baer et al. [9]: 60 months; Kan et al. [13]: 24–72 months). These limit understanding of long-term tissue stability and the natural history of IP levels around single-tooth implant restorations.
- Sixth, one included study (Khzam [26]) is gray literature (an unpublished master’s thesis) and one (Lédo et al. [12]) is available only as a conference abstract, both limiting the data that could be extracted and appraised.
- Finally, a further methodological consideration concerns the potential for overlapping patient cohorts. Multiple studies in this review originate from the same research groups using similar clinical protocols at the same institutions (Cosyn et al. 2011, 2012, and 2015 [5,6,11]; Meijndert et al. 2020 and 2021 [20,21]). It is possible that these represent partially overlapping patient populations, which could give disproportionate weight to the clinical approaches favored by these groups. The source publications do not provide sufficient patient-level detail to confirm or exclude overlap. Readers should be aware that clinical patterns attributed to multiple independent studies may partly reflect replicated findings from related cohorts rather than independent corroboration.

Future well-designed RCTs with adequate samples, pre-registered outcome definitions, blinded esthetic assessors, and follow-up of at least 3 years are needed to provide higher-certainty evidence for the clinical recommendations presented in this review.

A further scope-related limitation is that this review is confined to implant-based approaches to IP management. Strategies that may also influence interproximal tissue architecture in single-tooth edentulous spaces—including prosthetic IP simulation with ovate pontic design in fixed dental prostheses, subepithelial CTG around natural abutment teeth, and orthodontic extrusion of residual roots performed as a stand-alone IP regeneration strategy without subsequent implant placement—were not captured by the search strategy or inclusion criteria. The clinical literature on these approaches, though limited, may offer complementary perspectives on IP management that are relevant to cases in which implant placement is contraindicated or not preferred by the patient. This boundary should be considered when applying the findings of this review in broader clinical decision-making contexts.

### 4.8. Clinical Implications and Recommendations

The consistent patterns identified in this review support the following clinical guidance, understood as expert-level recommendations informed by predominantly observational evidence rather than established causal hierarchies.

- Standard-risk sites: Immediate implant placement with provisional restoration using a minimally invasive flapless approach—when anatomically indicated (intact buccal bone plate, contact point–crest distance ≤5 mm, adequate bone volume, and absence of active infection)—is the most extensively documented strategy and may reasonably be considered the primary clinical approach. Connective tissue grafting (CTG) should be considered as a supplementary measure in patients with a thin gingival phenotype (<1.0 mm), the anatomical factor most consistently associated with soft-tissue instability; the only available RCT addressing this comparison (Yoshino et al. [10]) provides preliminary controlled support for its benefit, without establishing it as a routine indication for all immediate protocols.
- High-risk anatomy: Sites presenting one or more of the following unfavorable conditions—thin gingival phenotype, reduced or dehisced buccal bone plate, contact point–crest distance >5 mm, severe vertical interdental tissue deficiency, or flattened papillary architecture resulting from prior tooth loss, trauma, or periodontal remodeling—carry a substantially elevated risk of esthetic shortfall and require individualized planning. At these sites, alveolar ridge preservation and contour augmentation should precede or accompany implant placement to optimize soft-tissue support. In cases where vertical tissue deficiency exceeds the compensatory capacity of soft-tissue grafting, orthodontic extrusion represents the approach associated with the largest papilla gains (approximately 4 mm, maintained over 24–72 months, Kan et al. [13]) and should be considered in carefully selected patients, with the acknowledgement that it requires longer treatment duration and biomechanical expertise.
- Patient communication: Esthetic perfection remains difficult to achieve consistently, and most patients attain Class I papilla (visible but incomplete fill) rather than Class 0 (complete fill to the contact point) as their best obtainable result. Pre-treatment communication about biological limitations, realistic esthetic expectations, and the potential for residual interdental embrasure space is an essential component of ethical management in the esthetic zone.

## 5. Conclusions

The management of IP loss in single-tooth implant-supported restorations within the esthetic zone is a clinically complex challenge for which high-certainty controlled evidence remains limited.

Across 27 predominantly observational studies, immediate implant placement with provisional restoration using minimally invasive flapless techniques, connective tissue grafting in thin gingival phenotypes, and maintenance of a contact point–crestal bone distance of ≤5 mm were the approaches most consistently associated with satisfactory IP fill, stable midfacial tissue levels, and high patient satisfaction. The two available RCTs provided preliminary support for CTG benefit and for the esthetic equivalence of delayed placement preceded by ridge preservation. Orthodontic extrusion was associated with near-complete IP reconstruction in cases of severe vertical tissue deficit. Available long-term evidence, limited to five observational studies, suggests that soft-tissue stability is largely maintained beyond the first year of function, though definitive conclusions await confirmation from controlled studies with standardized follow-up.

These findings represent consistent clinical associations from selected, protocol-adherent cohorts and should not be interpreted as established causal efficacy. Clinician judgment, patient-specific anatomy, and individualized risk-benefit assessment remain the primary guides to treatment selection until well-designed, adequately powered RCTs with pre-registered outcomes, blinded assessors, and a minimum three-year follow-up are available.

## Figures and Tables

**Table 1:** Pre-defined inclusion and exclusion criteria.

Criterion	Inclusion	Exclusion
Population	Adult patients (≥18 years) with single-tooth edentulism in the anterior maxilla (esthetic zone, incisors and canines)	Patients with posterior-only sites or multiple adjacent missing teeth without identifiable single-tooth component
Intervention	Surgical (immediate or delayed implant placement, flapless or flap-based), prosthetic, orthodontic, or combined procedures intended to maintain, restore, or regenerate the IP	Studies addressing implant survival/osseointegration exclusively, without reporting soft-tissue esthetic outcomes
Outcomes	At least one IP-related esthetic parameter: IP height, IP fill, IP index (e.g., Nordland–Tarnow classification), PES/WES, gingival margin stability, black triangle presence/absence, or patient-reported esthetic satisfaction	Studies reporting no IP-related or peri-implant soft-tissue esthetic outcome
Follow-up	Minimum 6 months post-restoration	Follow-up <6 months
Study design	Prospective or retrospective clinical studies, randomized controlled trials, comparative cohorts, longitudinal case series (*n* ≥ 6 patients)	Animal studies, in vitro studies, individual case reports (*n* < 6), systematic reviews, and meta-analyses (cited but not included in the primary evidence base)
Language	English	Non-English language publications

**Table 2:** Summary and critical appraisal of included studies evaluating IP preservation or regeneration at single-tooth sites in the esthetic zone.

Author (Year)	Study Design/OCEBM Level	Sample Size (*n*)/Follow-Up Period	Primary Intervention	Key Methodological Strengths	Key Methodological Limitations	Methodological Quality Rating (MQR)
Cabello et al. (2013) [1]	Prospective, longitudinal, multicenter/Level 4	14/12 months	Immediate, flapless, temporary restoration	Multicenter design; standardized trimodal protocol applied across sites; structured clinical measurements	Very small sample (*n* = 14); single arm; 12-month follow-up only; no comparator group	MQR-D
Choquet et al. (2001) [2]	Retrospective, comparative/Level 3b	26/6 months	Single implants, two uncovering techniques	Comparative design (two uncovering techniques); includes contact point–crest distance analysis; radiographic + clinical assessment	Retrospective design; high recall bias risk; very short follow-up (6 months); no blinding; dropout not reported	MQR-D
Belser et al. (2009) [3]	Cross-sectional, retrospective/Level 3b	45/24–48 months	Early implant placement, PES/WES	Larger sample (*n* = 45); validated PES/WES; multi-site patients; consecutive evaluation; structured esthetic assessment protocol	Retrospective cross-sectional design; no longitudinal within-patient change tracking; recall and selection bias	MQR-C
Tu et al. (2010) [4]	Prospective, longitudinal/Level 4	56/>6 months	Single implant, IP height/distance analysis	Largest sample in this review (*n* = 56); quantitative IP height measurement; contact point–crest distance analysis	Single arm; minimum follow-up only (>6 months); single center; non-standardized follow-up intervals	MQR-C
Cosyn et al. (2011) [5]	Prospective, longitudinal/Level 4	30/36 months	Immediate implant, minimal flap, grafting, temporary restoration	Prospective; 3-year follow-up (longest single-arm prospective in cohort); validated PES/WES; standardized surgical protocol	Single arm, no comparator; single center; no randomisation; moderate sample size only	MQR-C
Cosyn et al. (2012) [6]	Prospective, longitudinal/Level 4	22/12 months	Immediate implant, flapless, socket grafting, temporary restoration, CTG as needed	Prospective design; validated PES/WES outcomes; predefined surgical protocol; standardized data collection	Single arm, no comparator; small sample; single center; short follow-up; 12-month endpoint may not capture late tissue changes	MQR-D
Raes et al. (2011) [7]	Prospective, comparative, longitudinal/Level 2b	39/12 months	Immediate vs. conventional, with/without flap	Prospective comparative design (immediate vs. conventional); adequate sample; validated PES/WES; structured protocol	Non-randomized allocation; no blinding; 12-month endpoint; single center	MQR-C
Arora et al. (2017) [8]	Prospective, longitudinal/Level 4	30/24–60 months	Immediate, flapless, bone augmentation	Consecutive enrollment; long and heterogeneous follow-up range (2–5 years); standardized measurements; anterior maxilla only	Single arm; no comparator; variability in follow-up duration; single center	MQR-C
Baer et al. (2022) [9]	Prospective, longitudinal, multicenter/Level 2b	77/60 months	Immediate provisionalization on tapered conical implant	Largest sample (*n* = 77); multicenter design; longest follow-up (5 years); IP index + PES over time	Single arm; non-randomized; no control group; industry-sponsored design (potential bias)	MQR-C
Yoshino et al. (2014) [10]	Prospective, RCT, longitudinal/Level 1b	20/12 months	Immediate implant, CTG vs. no graft	Randomized controlled design; direct comparison CTG vs. no graft; blinded outcome assessor; defined endpoints	Very small sample per group (*n* = 10/arm); single center; single assessor; 12-month follow-up; underpowered for subgroup effects	MQR-B
Cosyn et al. (2015) [11]	Prospective, longitudinal/Level 3b	50/12 months	Ridge preservation, contour augmentation, CTG	Largest prospective single-arm cohort (*n* = 50); consecutive enrollment; defined ridge preservation and augmentation protocol	Single arm; 12-month follow-up; no comparator; single center	MQR-C
Lédo et al. (2018) [12]	Prospective, comparative, longitudinal/Level 3b	20/12 months	Immediate implant, autogenous vs. synthetic graft	Prospective comparative design (autogenous vs. synthetic graft); 12-month IP score assessment	Published as conference proceedings (limited extractable data); small sample; 12-month follow-up; allocation not randomized	MQR-D
Kan et al. (2023) [13]	Retrospective, longitudinal/Level 4	14/48.4 months	Orthodontic extrusion for IP regeneration	Long follow-up (mean 48.4 months; range 24–72 months); unique orthodontic extrusion technique assessment	Retrospective; very small sample (*n* = 14); no control group; selection bias; loss-to-follow-up not reported	MQR-D
Cardaropoli et al. (2022) [15]	Comparative, longitudinal/Level 1b	48/12 months	Immediate vs. delayed after ridge preservation	Randomized design; adequate sample; validated PES/WES + clinical outcomes; controlled comparison (immediate vs. delayed after ridge preservation)	12-month follow-up only; blinding of clinical evaluators not reported; single center	MQR-B
Cardaropoli et al. (2005) [16]	Prospective, longitudinal/Level 4	11/18 months	Two-stage Brånemark, delayed loading	Prospective; 18-month follow-up; structured Brånemark two-stage protocol; sequential tissue assessment	Very small sample (*n* = 11); single arm; high risk of type II error; single center	MQR-D
De Rouck et al. (2008) [17]	Longitudinal, consecutive/Level 4	30/12 months	Immediate, flap, grafting, screw-retained temporary	Consecutive enrollment; validated PES/WES; standardized immediate implant + provisional protocol; structured assessments	Single arm; 12-month follow-up; no comparator group; single center	MQR-C
Chou et al. (2012) [18]	Comparative, longitudinal/Level 3b	30/6 months	Single implant, gingival phenotype comparison	Gingival phenotype comparison (thin vs. thick); standardized IP alteration assessment; adequate group sizes	Very short follow-up (6 months only); no IP height quantification; non-randomized gingival phenotype allocation	MQR-D
Mohamed et al. (2024) [19]	Prospective, longitudinal/Level 4	10/6 months	Delayed implant, modified pedicle CTG for IP	Specific pedicle CTG technique for delayed implants; bilateral IP height measurements	Very small sample (*n* = 10); shortest follow-up (6 months); single arm; single center; underpowered	MQR-D
Meijndert et al. (2020) [20]	Prospective, longitudinal/Level 4	60/16 months	Bone level tapered implant, immediate/late, augmentation	Adequate sample (*n* = 60); bone level tapered implant–specific; PES/WES and patient-reported outcomes	Single arm; 16-month follow-up; no comparator; single center	MQR-C
Meijndert et al. (2021) [21]	Prospective, longitudinal/Level 4	30/≥6 months	Ridge preservation, delayed implant, temporary restoration	Ridge preservation + delayed implant protocol; VAS patient satisfaction reported; PES/WES outcomes	Single arm; minimum 6-month threshold; moderate sample; single center	MQR-C
Palattella et al. (2008) [22]	Prospective, comparative, longitudinal/Level 2b	16/2 years	Immediate vs. delayed placement, immediate restoration	Prospective; 2-year follow-up; comparative design (immediate replacement vs. immediate restoration)	Very small sample per group; non-randomized; potential selection bias in group allocation	MQR-C
Tortamano et al. (2010) [23]	Prospective, longitudinal/Level 4	12/18 months	Immediate implant, temporary restoration, central incisor	Prospective; 18-month follow-up; structured clinical and radiographic outcomes	Very small sample (*n* = 12); single arm; single center; underpowered; highly selected (central incisor only)	MQR-D
Khzam (2010) [24]	Retrospective, longitudinal/Level 4	34/12–27 months	Immediate, flapless, screw-retained temporary	Adequate sample (*n* = 34); varied follow-up duration (up to 27 months)	Gray literature (unpublished thesis, not peer-reviewed in standard sense); retrospective; no comparator; selection bias uncontrolled	MQR-D
Todescan et al. (2023) [25]	Prospective, longitudinal/Level 4	26/12 months	Immediate implant, definitive abutment, temporary restoration	Recent (2023); prospective; standardized immediate abutment protocol; radiographic and clinical endpoints	Single arm; small sample; 12-month follow-up; no control group; single center	MQR-D
Amato et al. (2022) [26]	Longitudinal, observational/Level 4	10/24–60 months	Flapless, subcrestal implant, CTG, bone graft, membrane	Long follow-up range (2–5 years); specific dehiscence site protocol; PES outcome reporting	Very small sample (*n* = 10); observational; no control group; high risk of selection bias; underpowered	MQR-D
Chang et al. (1999) [27]	Comparative, longitudinal/Level 2b	20/≥6 months	Implant vs. contralateral tooth, soft tissue/crown	Contralateral natural tooth as internal comparator; combined clinical and digital soft-tissue analysis	Small sample; non-randomized; minimal follow-up threshold (≥6 months); consecutive enrollment not stated	MQR-C
Chen et al. (2023) [28]	Retrospective, case series, longitudinal/Level 4	15/24–36 months	Bone ring technique, simultaneous implant	Specific bone ring technique evaluated; mid-term follow-up (2–3 years); combined clinical and radiographic assessment	Retrospective; small sample (*n* = 15); single arm; no control group; selection bias	MQR-D

The table outlines the main characteristics of the studies included in the review, detailing the author, year, study design, sample size, follow-up period, and primary intervention. Interventions include various combinations of immediate or delayed implant placement, flapless or minimal-flap surgery, CTG, ridge preservation, and different loading protocols, all aimed at maintaining or regenerating the interdental papilla. Critical appraisal of the 27 studies was included.

**Table 3:** Esthetic outcomes, complications, and patient satisfaction across the included studies.

Author (Year)	Follow-Up	Esthetic Outcomes	Complications/Failures	Patient Satisfaction
Cardaropoli et al. (2005) [16]	18 months	IP fill ≥50% in 86%; labial margin shift	None major reported	Not reported
Cosyn et al. (2012) [6]	12 months	PES = 12.15; distal IP lower than 0.5 mm	Minimal midfacial recession in thin gingival phenotype	High
Cabello et al. (2013) [1]	12 months	Stable buccal margin; recession 0.45 mm	None major	Not reported
Choquet et al. (2001) [2]	6 months	IP fill nearly complete if ≤5 mm contact–crest	Not reported	Not reported
Chou (2012) [18]	6 months	Thin gingival phenotype: more IP alteration; survival 100%	None	Not reported
Yoshino et al. (2014) [10]	12 months	CTG vs. no graft: −0.25 vs. −0.70 mm midfacial; IP index > 50% in 75–80%	None	High
Raes et al. (2011) [7]	12 months	24% esthetic failures (PES < 8/WES < 6)	8% complications	Moderate
Todescan et al. (2023) [25]	12 months	Minimal IP loss (−0.04 to −0.09 mm); facial level −0.13 mm	None	Not reported
Meijndert et al. (2020) [20]	16 months	Median PES = 6, WES = 8	No adverse events	Not reported
Meijndert et al. (2021) [21]	12 months	Median PES = 7, WES = 8	Stable	VAS 86.6%
Palattella et al. (2008) [22]	24 months	Immediate vs. delayed: no significant difference	None	Not reported
Cardaropoli et al. (2022) [15]	12 months	Stable esthetics across groups	None	Not reported
Arora et al. (2017) [8]	24–60 months	Soft-tissue loss 0.06 mm; midfacial 0.29 mm	None	Not reported
Amato et al. (2022) [26]	24–60 months	PES = 12.5; stable esthetics	None	High
Baer et al. (2022) [9]	60 months	90.1% optimal IP index; PES improved	Minimal plaque/bleeding	Improved over time
Kan et al. (2023) [13]	24–72 months	IP gain = 4 mm; stable	None	High
De Rouck et al. (2008) [17]	12 months	PES/WES improved	Minimal recession	93% satisfaction
Mohamed et al. (2024) [19]	6 months	Mesial/distal IP significantly increased	None	Not reported

This table summarizes the main findings of the reviewed studies regarding esthetic outcomes, complications or failures, and patient satisfaction. Most studies reported stable or improved papilla fill and soft-tissue levels following immediate or early implant placement, with low complication rates and high esthetic satisfaction, particularly when CTG or soft-tissue augmentation techniques were applied.

## Data Availability

The data that support the findings of this study are available from the corresponding author upon reasonable request.
